# Modulating the Crosstalk between the Tumor and Its Microenvironment Using RNA Interference: A Treatment Strategy for Hepatocellular Carcinoma

**DOI:** 10.3390/ijms21155250

**Published:** 2020-07-24

**Authors:** Mariam Mroweh, Thomas Decaens, Patrice N Marche, Zuzana Macek Jilkova, Flora Clément

**Affiliations:** 1 Institute for Advanced Biosciences, Research Center Inserm U 1209/CNRS 5309, 38700 La Tronche, France; mariam.mroweh@gmail.com (M.M.); tdecaens@chu-grenoble.fr (T.D.); patrice.marche@inserm.fr (P.N.M.); 2 Université Grenoble Alpes, 38000 Grenoble, France; 3 Laboratory of Cancer Biology and Molecular Immunology, Faculty of Sciences I, Lebanese University, Hadath Beirut 6573-14, Lebanon; 4 Service d’hépato-Gastroentérologie, Pôle Digidune, CHU Grenoble Alpes, 38700 La Tronche, France

**Keywords:** HCC, tumor microenvironment, RNAi, miRNA, shRNA, siRNA

## Abstract

Hepatocellular carcinoma (HCC) is the most common primary liver malignancy with one of the highest mortality rates among solid cancers. It develops almost exclusively in the background of chronic liver inflammation, which can be caused by viral hepatitis, chronic alcohol consumption or an unhealthy diet. Chronic inflammation deregulates the innate and adaptive immune responses that contribute to the proliferation, survival and migration of tumor cells. The continuous communication between the tumor and its microenvironment components serves as the overriding force of the tumor against the body’s defenses. The importance of this crosstalk between the tumor microenvironment and immune cells in the process of hepatocarcinogenesis has been shown, and therapeutic strategies modulating this communication have improved the outcomes of patients with liver cancer. To target this communication, an RNA interference (RNAi)-based approach can be used, an innovative and promising strategy that can disrupt the crosstalk at the transcriptomic level. Moreover, RNAi offers the advantage of specificity in comparison to the treatments currently used for HCC in clinics. In this review, we will provide the recent data pertaining to the modulation of a tumor and its microenvironment by using RNAi and its potential for therapeutic intervention in HCC.

## 1. Hepatocellular Carcinoma

Hepatocellular carcinoma (HCC) is the most common type of liver malignancy, comprising 75–85% of all liver cancers. It is a leading cause of cancer-related death, being estimated to be the fourth most common cause of cancer-related death overall worldwide [1]. The emergence of HCC is triggered by a chronic inflammation background. The typical stages of emergence can be summarized as follows: inflammation, fibrosis, cirrhosis and finally HCC. The risk factors implicated in favoring HCC, mainly through the induction of chronic inflammation, include viral hepatitis C (HCV), viral hepatitis B (HBV), chronic alcohol intake, metabolic disorders leading to non-alcoholic fatty liver diseases and non-alcoholic steatohepatitis (NASH), the consumption of toxins (such as aflatoxins) and hereditary diseases such as hemochromatosis [2]. The main problems that HCC treatment is facing are the delays in the diagnosis, recurrence and drug resistance. Some patients remain asymptomatic until the advanced stages of the disease; however, if detected at an early stage, HCC can be curable by surgical resection, percutaneous ablation or liver transplantation. Unfortunately, at an advanced stage, the treatment options are limited. The current standard therapy option for advanced HCC treatment is Sorafenib [3]. Additionally, Lenvatinib (another first-line drug for treating HCC besides Sorafenib), Regorafenib and Cabozantinib, Nivolumab, Pembrolizumab and Atezolizumab plus Bevacizumab (second-line treatment after Sorafenib) were recently approved for HCC treatment [4], but these drugs show no additional improvement in survival compared to Sorafenib [5]. Thus, the search for a possible treatment with better efficacy continues, and currently, various immunotherapies and angiogenic inhibitors are under testing as candidates for HCC treatment, targeting the complex intrahepatic network. Noticeably, the search for a drug shifted from the conditional “direct tumor-targeting” tyrosine kinases to “neighboring effectors” targeting the immune and angiogenic network of the tumor. From this shift, one can infer that the players in HCC extend beyond the tumor into the tumor microenvironment and thus serve as certified targets for treatment [2]. Very recently, a new therapeutic option was published demonstrating the superiority of Atezolizumab—an anti-programmed death-ligand 1 (PDL-1) antibody—plus Bevacizumab, an anti-vascular endothelial growth factor (VEGF) antibody, compared to Sorafenib in the first line treatment of advanced HCC, and the FDA approved this treatment on 29 May 2020 [6].

## 2. RNA Interference (RNAi) Overview

The Nobel Prize in Physiology and Medicine was awarded in 2006 to RNAi discoverers, Andrew Z. Fire and Craig C. Mello [7]. RNAi involves a double-stranded ribonucleic acid that interferes with a specific messenger RNA (mRNA) and prevents its translation or leads to its degradation, therefore decreasing the expression levels of the corresponding protein. This mechanism censors the output of the genome and dictates the fate of a specific transcript, inhibiting its translation into the final active form, the protein. Due to the aberrant activities of a variety of proteins in cancers, RNAi represents a promising tool in cancer therapy. Moreover, as RNAi interrupts the gene of interest (and can be specific to a mutated version of a gene), mRNA, and protein flow, it can modulate the secretory profile of the cells, leading to disrupted crosstalk, which is essential for tumor progression [8].

**Figure 1:**
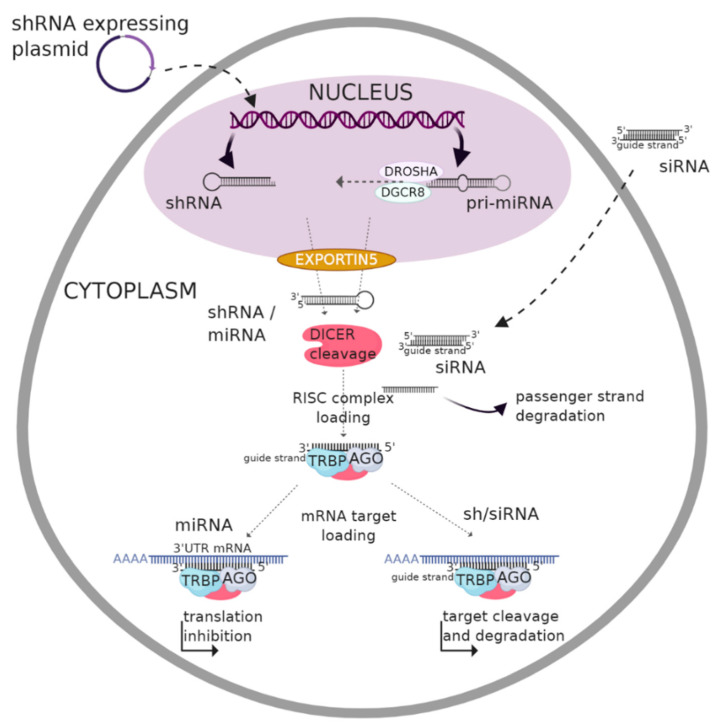
Scheme of three different RNA interference (RNAi) pathways: micro(mi)RNA, short hairpin (sh)RNA and small interfering (si)RNA. shRNA and miRNA mediated silencing are based on a modification of the genomic content within the nucleus whereas siRNAs act directly in the cytoplasm of cells and do not require nuclear import. shRNAs and miRNAs are introduced into the cell in the form of DNA, and it is essential that they are transported into the nucleus to be transcribed into dsRNAs with a hairpin-like structure and are thus termed pri-shRNAs and pri-miRNAs, respectively, which are further cleaved in the nucleus by the DROSHA enzyme. The resulting transcripts are termed pre-shRNAs and pre-miRNAs, respectively, and are exported to the cytoplasm via Exportin 5. siRNAs, on the other hand, are introduced in the form of dsRNA sequences and are not delimited by a nuclear transport step; they are cleaved by DICER enzyme to attain their final form. In the cytoplasm, the three pathways converge as they are all loaded to the RNA induced silencing (RISC) complex. After that, they diverge again in their mechanism of action to silence a target gene: a miRNA-RISC complex inhibits translation, whereas sh/siRNA-RISC complexes bind to mRNA sequencesresulting in their degradation.

RNAi is a regulatory mechanism involving small regulatory RNAs (belonging to non-coding RNAs—ncRNA) that are not translated into proteins. The RNAi mechanism is one way of inducing post-transcriptional gene silencing and can participate as a natural process of resistance to the presence of pathogenic exogenous double-stranded RNA (dsRNA) [9]. The presence of dsRNA in the cell is termed to be “abnormal”, and it is degraded by the cellular machinery upon recognition through Toll-like receptor (TLR) activities. dsRNAs can be of viral origin but can also be from endogenous genes such as transposons [10]. Therefore, by mimicking this process, i.e., the introduction of RNA molecules with the ability to bind to the target mRNA and thus labelling it for degradation, it is possible to control the expression of specific genes for treatment purposes. Diverse studies have shown, in vitro, the efficiency of this mechanism in numerous pathologies, specifically in inflammatory diseases as well as cancer [11,12]. RNAi comprises three different types of RNA molecules: microRNAs (miRNA), short hairpin RNAs (shRNA) and small interfering RNAs (siRNA) (Figure 1).

### 2.1. Biogenesis and Gene Silencing Mechanism of MicroRNA

A miRNA is an endogenous single-stranded RNA molecule of 19–22 nucleotides (nt) derived from a double-stranded region of a 60–70 nt RNA hairpin precursor. Sequences encoding miRNAs are dispersed in the genome and occur in the form of clusters. They can be intercalated between two genes, i.e., in intergenic regions, or within the intron sites of a specific gene [13]. The biogenesis of miRNAs takes place in both the nucleus and cytoplasm. Depending on the DNA region, the miRNA gene is transcribed by either RNA polymerase II or III (generally the latter). The resulting primary (pri-)miRNA is then processed to give the precursor (pre-)miRNA, which assumes a loop structure and is exported into the cytoplasm by the help of Exportin-5. Further maturation occurs in the cytosol to produce a mature miRNA that binds to the target mRNA. The silencing of the target mRNA is initiated by a partial hybridization of the miRNA-RNA induced silencing complex (RISC) on the 3′-untranslated region of the mRNA through base pairing [14]. The induction of miRNA, therefore, implies nucleus modification.

### 2.2. Biogenesis and Gene Silencing Mechanism of shRNA

Another form of RNAi is shRNA. shRNAs are engineered RNA sequences that can be produced within the cell from a DNA vector. The transfection of DNA that expresses shRNA into the nucleus of the target cell can be achieved by using different vectors such as lentivirus, adenovirus, plasmids, polymerase chain reaction amplicons or small circular DNA sequences [15,16]. The first transcripts of shRNAs known as pri-shRNAs with hairpin-like structures are transcribed within the nucleus [17] and then processed to give pre-shRNAs of 50–70 nt in length, which are then exported to the cytosol through Exportin-5. After cytosolic maturation, the pre-shRNA is then cleaved to generate double-stranded fragments of 21–22 nt shRNAs. Finally, the silencing mechanism is similar to that of siRNAs, as described below [15]. The strong expression of shRNA may be less beneficial as it can alter the endogenous RNAi pathway, thus increasing the saturation of the miRNA pathway. In addition to the off-targets, the lack of an efficient delivery system poses a major challenge for shRNA-based therapy [18]. Additionally, the nonspecific binding of RNAi molecules to cellular components, such as non-targeted mRNAs, can increase the immune- and toxicity-related responses. Moreover, the use of viral vectors for delivery forces a margin of toxicity, insertional mutagenesis and immunogenicity, thus limiting their clinical application [18].

### 2.3. Biogenesis and Gene Silencing Mechanism of siRNA

RNAi technology has evolved over time, with the easiest approach being delivering chemically synthetized siRNAs. Primarily, siRNAs were thought to be exogenous in origin; however, several studies have identified endogenous siRNAs arising from genomic loci [19]. siRNAs are conserved among eukaryotes and involved in gene expression regulation and cell proliferation [8,20]. siRNAs are produced from a long double-stranded RNA (200–500 bp) precursor that is cleaved by Dicer via two RNAse III domains. The resulting dsRNA fragments are unwound by helicase enzymes, and the antisense strand known as the guide strand is associated with a RISC to form a siRNA-RISC. This complex is then directed to the target mRNA, and the cleavage of the latter is induced by the active site residues in the P-element Induced Wimpy testis (PIWI) domain of the argonaute (AGO) protein [21].

## 3. Clinical Application of RNAi

Despite the recent advances in the clinical application of RNAi, the avoidance of nonspecific toxicity is a major critical challenge in the development of RNAi therapeutics. The toxicity and activity of RNAi drugs depend largely on sequences present or absent in the transcriptome. Thus, a siRNA that has no off-target effects in rodents could induce intolerable off-target-related toxicity in humans, and a siRNA that induces good silencing in animals may have insufficient activity against the human gene. Therefore, a careful preclinical development process is required. Another important issue in RNAi-based therapeutics is the possible dose-limiting toxicity of carriers, related to their heterogeneity in terms of complexity, uniformity and stability, as reviewed recently [9].

Unlike gene therapy, which is based on the modification of the genomic content within the nucleus, siRNAs act directly in the cytoplasm of cells and do not require nuclear import. Therefore, siRNA-based therapies raise a much lower threat of introducing mutations that can lead to cancer. The first RNAi-based therapeutics have been introduced into clinical practice recently. Indeed, Patisiran, the first-ever therapeutic based on RNAi, was approved by the FDA in August 2018. This siRNA specifically blocks the expression of an abnormal form of the transthyretin protein in patients with hereditary transthyretin amyloidosis [22]. The next-ever drug that was developed based on RNAi, Givlaari, was approved by the FDA in November 2019 for the treatment of acute hepatic porphyria [23]. Both drugs were developed by Alnylam Pharmaceuticals, the RNAi pioneer company that managed to encase its synthetic siRNA in a lipid-based nanoparticle and deliver it into the liver. Moreover, many studies have validated the use of siRNA to treat various viral infections, including Ebola, in non-human primates in a very specific way, as siRNAs can be tailored for each epidemic strain [24]. These studies highlight the therapeutic potential of siRNAs, a potential that is currently exploited to propose innovative solutions to target the crosstalk between the tumor cell and its environment.

### 3.1. RNAi and Cancer

To date, there is no RNAi-based drug approved for anticancer therapy, but several therapeutics are currently undergoing clinical trials. Originally, RNAi-based anticancer therapy was proposed to target oncogenes. In fact, proto-oncogene activation can occur through genetic alterations including gene fusion, translocation, mutation or chromosomal rearrangement [25]. Therefore, an imbalance between proto-oncogenes and tumor suppressor genes can sustain cancer development [26]. RNAi could be used to inhibit the mRNA translation of oncogenic genes and improve chemotherapy efficiency by reducing the activity of multidrug resistance-related genes within cancer cells [27,28]. However, there are still major barriers to the systemic delivery of these macromolecules to target cells. For instance, the plasma membrane remains the major barrier to RNAi molecules due to its hydrophilic nature, global negative charge and high molecular weight, which reduces its uptake efficiency. In addition, the specificity of gene targeting, intracellular enzymatic degradation, stability and the kinetics of RNAi molecules in circulation and their local distribution are among the important parameters to be developed and optimized [29,30]. Recently, non-viral vectors, especially nanoparticle carriers such as polymers (polyethylene glycol, polyethylenimine (PEI), poly l-lysine and chitosan), lipids (lipid nanoparticles, liposomes, micelles, etc.) or inorganic nanoparticles (carbon nanotubes, gold and mesoporous silica nanoparticles), have shown multiple advantages concerning RNAi delivery [31]. In fact, nanoparticles have received considerable attention due to their ability to protect RNAi molecules from enzymatic degradation and their capacity to be associated with specific peptides, increasing their selectivity for tumor cells [32,33]. In addition, chemical modifications involving sugar and phosphate and chemical structure modifications in the oligonucleotide backbone of RNAi have been proven to be efficient as they considerably reduce toxicity, off-target effects and degradation by nucleases [34,35]. Clinical trials of RNAi-based therapeutics for solid tumors including HCC are listed in Table 1.

### 3.2. RNAi-Mediated Therapeutic Intervention in the Context of HCC

The potential anticancer effects of RNAi technology show that the gene silencing of overexpressed genes in tumor cells—involved in tumor growth, proliferation, signaling pathways, drug resistance or tumor metastasis—could provide curative benefits and reduce HCC development [36]. Moreover, RNAi could target other cells in the liver and thereby modify the crosstalk between the tumor and its surrounding microenvironment to limit the potentiation effects of either of the cells on each other [37].

The use of RNAi in treating HCC comprises the RNAi molecule and its vector, and the success of such an intervention is dependent on the efficiency of the delivery system along with the RNAi efficiency, e.g., many studies have been done on the carrier to maximize the siRNA delivery. For instance, a study showed that the lipidation of a PEI-based polyplex increases the stability of the complex in vivo and results in better accumulation in the liver and knockdown in orthotopic HCC xenografts [38]. A clinical trial, NCT01591356, involving siRNA-mediated therapy in solid tumors is currently ongoing for HCC patients. It was launched by the MD Anderson Cancer Center and is expected to be completed by June 2020. This trial evaluates the effect of ephrin type-A receptor 2 (EphA2)-targeting siRNA in patients with advanced or recurrent solid tumors using neutral liposomal delivery [39]. Furthermore, the FDA granted in 2020 an orphan drug designation to the therapeutic candidate, STP705, a siRNA targeting both the transforming growth factor (TGF)-β and cycloxygenase-2 genes for the treatment of HCC [40].

## 4. Tackling the Crosstalk between the Tumor and Its Microenvironment Using RNA Interference

**Figure 2:**
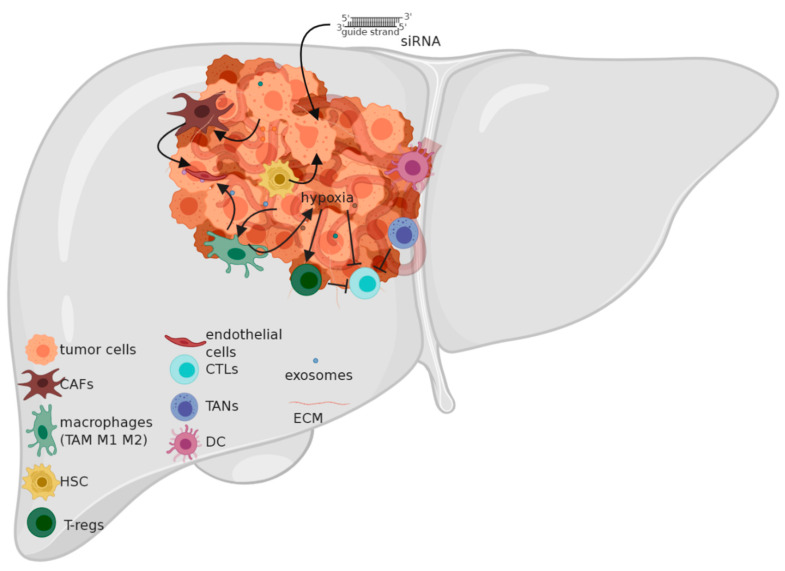
Tumor microenvironment in hepatocellular carcinoma (HCC). CAFs: Cancer associated fibroblasts; TAM: tumor associated macrophages; HSC: hepatic stellate cell; T-regs: T-regulatory cells; CTLs: cytotoxic T-Lymphocytes; TANs: tumor associated neutrophils; DC: dendritic cell; ECM: extracellular matrix; siRNA: small interfering ribonucleic acid. The cellular and non-cellular components of the tumor microenvironment of HCC are modulated by the tumor–tumor microenvironment communication and help the cancer to thrive by reciprocal communication. siRNAs can serve as a communication barrier by targeting the effector molecules exchanged. CAFs arise from resident cells in the tumor microenvironment and support tumor growth, survival and metastasis. TAMs are a macrophage subset that exhibits a tumor-supporting role. HSCs are modulated by the tumor to sustain its invasiveness and growth. T-regs depotentiate the immune response against the tumor. CTLs aim to combat the tumor but are often depotentiated. TANs recruit TAMs and T-regs to the tumor’s vicinity. DCs impair the T-cell response against the tumor. The ECM acts as a mediator of communication between the various cells. Exosomes shuttle effector molecules (proteins and/or nucleic acids) between the tumor and the cell of origin and vice versa.

The tumor microenvironment is a crucial player in the progression of the tumors in all cancers, and HCC is no exception. The key to cancer progression and thriving is the presence of a milieu that sustains the cancer’s multiplication and helps in combating the various stresses that come its way; among them are hypoxia and the immune response that attempts to combat the cancer. The microenvironment status is a key player in evading these stresses and creating coping mechanisms for the cancer to thrive. As explained above, inflammation drives HCC development. This suggests the key role of immune cells in the vicinity of the tumor before the emergence of HCC. A change in the status of the tumor microenvironment from a “combating” one to a “permissive” one is crucial. This is established by changes in the tumor microenvironment of the liver including many effectors: extracellular matrix (ECM) components, cancer-associated fibroblasts (CAFs), endothelial cells, hepatic stellate cells (HSCs) and immune cells (migratory and resident) (Figure 2). All of these mediate a crosstalk with the tumor via a profile of secretome ranging from chemokines, cytokines and growth factors to extracellular vesicles (e.g., exosomes) harboring effector molecules (nucleic acids and/or proteins) that aid in the tumor progression and the conversion of the milieu into a pro-tumorigenic one. This back-and-forth communication serves as a feedback loop to ensure a sustained adaptation according to the tumor status [41].

### 4.1. Targeting Communication with Extracellular Components

#### 4.1.1. Extracellular Matrix (ECM)

The ECM is a mesh of an interstitial matrix and basal membrane composed of proteoglycans and hyaluronic acid that hold the organ together. However, this mesh is not only a matrix witnessing the evolution of the tumor in HCC; rather, it facilitates the interactions between the affectors and their corresponding effectors. It acts as a bridge in the crosstalk between the tumor and the different cells in the tumor‘s vicinity and faces many changes due to hepatocarcinogenesis [42].

Heparin sulfate (HS) plays an important role in HCC and mediates the binding of the growth factors to their respective receptor tyrosine kinases. Heparin-degrading endosulfatases sulfatase 1 and sulfatase 2 in the ECM regulate such a process [43]. As crosstalk mediators and potentiators of HCC, RNAi approaches have been developed to target the different growth factors in the vicinity of HCC.

Besides heparin sulfate, a heparan sulphate proteoglycan glypican 3 (GPC3) is located at the extracellular side of the cell membrane through a glycosylphosphatidylinositol anchor. It has recently emerged as a potential biomarker and/or therapeutic target for HCC. Silencing GPC3 in hepatocytes from proximal liver tissue and human HCC cells, with a targeted siRNA, was shown to decrease proliferation and boost apoptosis, along with a decrease in the invasive profile [44]. Furthermore, another study exploited the synergistic effect of the siRNA-mediated knockdown of GPC3 along with Sorafenib to combat HCC. Liposomes harboring GPC3 siRNA along with Sorafenib were delivered to HepG2 cells. The results showed a hindered proliferation, possibly due to the decrease in Cyclin D1 expression following GPC3 knockdown and Sorafenib administration, along with an increase in the apoptosis rate. In vivo subcutaneous xenografts of HepG2 cells in nude mice yielded results consistent with those obtained in vitro [45].

Heparanase, another important component of the ECM, plays a prominent role in tumorigenesis [46]. It contributes to the cleavage of the HS side chains of heparin sulfate proteoglycans, releasing sequestered bioactive molecules. In the context of HCC, heparanase has been shown to promote metastasis by two means: the degradation of the ECM components and a non-enzymatic alteration of the adhesive properties of HCC [47]. Reducing the heparanase expression utilizing siRNA could efficiently inhibit tumor metastasis [48].

Growth factors such as VEGF and hepatocyte growth factor (HGF), and epidermal growth factor receptor (EGFR) have been observed to be overexpressed in many cancers including HCC, as they play a crucial role in different mechanisms involving tumor proliferation, metastasis and angiogenesis [49,50,51]. A shRNA targeting VEGF was designed to inhibit VEGF expression in HCC cells and liver tumor tissues. The latter was administrated intratumorally or via intravenous injection into orthotopic allograft liver tumor-bearing mice. The results demonstrated a more effective suppression of tumor angiogenesis and tumor growth in the different HCC models studied [52]. Moreover, a study designed a multiple targeting siRNA which could simultaneously suppress three genes: NET-1, EMS1 and VEGF. This co-silencing reduced tumor proliferation and growth and induced tumor cell death [53,54]. Similarly, the simultaneous silencing of VEGF and KSP using a siRNA cocktail in Hep3B cells inhibited cell proliferation, migration and invasion and also promoted tumor apoptosis [48]. In another study, a siRNA was used to suppress migration inhibitory factor (MIF) cytokines that play an important role in HCC proliferation. Upon MIF knockdown, the tumor growth rate was reduced in both HCC cell lines and in an in vivo xenograft model, along with an increased expression of apoptosis-related proteins [55]. As growth factors have an impact on the proliferation of HCC, RNAi targeting proliferation mediators in HCC have been utilized. Cyclin E and cyclin-dependent kinase 2 are essential actors in the cell cycle and initiation of DNA replication [56], and overexpression of cyclin E has been found in 70% of HCC cases. Accordingly, a siRNA targeting the coding region of cyclin E was designed, this showed a suppression of cyclin E expression up to 90% in HCC cell lines and also inhibited HCC tumor growth in nude mice [57].

Another group of matricellular proteins involved in HCC is cellular communication network factors (CCN), core regulatory proteins—including cysteine-rich angiogenic protein 61 (CYR61 or CCN1), connective tissue growth factor (CTGF or CCN2), nephroblastoma overexpressed (NOV or CCN3) and Wnt1-Inducible Signaling pathway proteins (WISP-1 or CCN4)—that modulate cell–matrix interactions to modify the cellular phenotype. CCNs play a role in differentiation, adhesion, migration, mitogenesis, chemotaxis and angiogenesis. The first four members of this family are shown to play a role in HCC: CCN1 to 4. CCNs induce the secretion of chemokines and cytokines to establish an inflammatory milieu and orchestrate the recruitment of immune cells to the tumor microenvironment. For instance, platelet-derived CCN1 increases the percentage of reactive oxygen species in hepatocytes, leading to macrophage activation and immunosuppression. CCN1 has also been shown to increase the expression of several proinflammatory signals through the activation of integrin–nuclear factor κB (NFκB) signaling in macrophages [58,59]. Moreover, fibronectin, laminin, collagen and elastin in the ECM are involved in chemotaxis and cell–cell interactions. Additionally, the laminin-5-dependent overexpression of integrins α3β1 and α6β4 positively correlates with the invasive and metastatic potentials of HCC cells. Changes are introduced in the adhesive and migratory characteristics of HCC cells by the α3β1 integrin by the mediation of the interaction between the ECM and the cells [60]. Silencing β1 and αv integrin subunits by the nanoparticle delivery of siRNAs in mouse liver reduced tumor proliferation and increased tumor cell death without harming the healthy liver tissue [61].

#### 4.1.2. Extracellular Vesicles (Exosomes)

Exosomes are small membrane vesicles that are generated by many different cell types, in both normal and pathological conditions. These small nanoparticles are released by the fusion of multivesicular bodies with the plasma membrane [62]. Their role in crosstalk lies in the cargo they carry within them. Exosomes play important roles in the exchange of biological information as substance transport carriers and in the regulation of the cellular microenvironment by delivering a variety of biological molecules, including mRNAs, miRNAs and proteins. The shuttling of exosomes between the different effectors has been shown to reshape the tumor microenvironment in order to support carcinogenesis. Among the effects of exosomes are the suppression of the immune response, the favoring of angiogenesis, the remodeling of the ECM and changes in the stromal cells [63,64,65]. Exosomes have been shown to transfer genetic material from cancerous cells to normal ones, mediating tumor progression, traverse the blood stream to distant areas to elicit a metastatic site for the tumor, and modulate the anti-tumor immunity. On the level of the nucleic acid content of exosomes, extensive research has been done on the miRNA of HCC-derived exosomes. Studies over the years have provided a panel of miRs that are transported from the tumor to the adjacent cells, some of which are as follows: miR-584, miR-517c, miR-378, miR-520f, miR-142-5p, miR-451, miR-518d, miR-215, miR-376a, miR-133b and miR-367, miR-21, miR-192, miR-221, miR-122, miR-423-5p, miR-21-5, plet-7d-5p, let-7b-5p, let-7c-5p, miR-486-5p and miR-10b-5p, miR-519d and miR-1228. The different miRNAs modulate the gene expression in the recipient cells, favoring metastasis, tumor progression, drug resistance and recurrence [66]. Techniques to exploit miRNA to tackle the crosstalk between tumor cells and their environment have recently emerged. For instance, miR-320a, which is downregulated in several cancers, was found to inhibit c-Myc expression in HCC tissues and cell lines. Therefore, the upregulation of miR-320a by transfecting the cells with miR-320a mimics inhibited tumor proliferation and invasion by decreasing the expression of c-Myc in HCC cells [67].

A study in 2016 described that exosomes alter drug sensitivity by releasing molecules such as mRNAs and miRNAs into neighboring cells. This study sheds the light on the activation of the HGF/c-Met/Akt signaling pathway via HCC-cell derived exosomes resulting in the inhibition of Sorafenib-induced apoptosis, thus emphasizing the role of exosomes in Sorafenib resistance in liver cancer [68]. Wang and colleagues in 2018 described that HCC cell line HepG2-derived exosomes could be actively internalized by adipocytes differentiated from mesenchymal stem cells (MSCs) and caused significant transcriptomic alterations, in particular induced inflammatory phenotypes in adipocytes [69]. Additionally, the 14-3-3ζ protein high expression in CD8^+^ T cells induces their exhaustion. This was concluded by a comparative assessment of the expression level of programmed cell death protein-1 (PD-1), T-cell immunoglobulin and mucin domain-containing molecule-3 (TIM-3), lymphocyte-activation gene 3 (LAG3) and cytotoxic T-lymphocyte-associated protein-4 (CTLA-4) between 14-3-3ζ^high^ and 14-3-3ζ^low^ CD8^+^ T cells. Tumor-derived exosomes mediate the transfer of the 14-3-3ζ protein to the tumor-infiltrating lymphocytes, leading to their exhaustion and/or their differentiation into T-regulatory cells (T-regs) [70].

Aside from miRNAs, exosomes have been shown to shuttle mRNAs and long ncRNAs. Many studies have shown that HCC cell lines with a great metastatic potential secrete exosomes carrying proto-oncogenes, in the form of mRNA, such as Met S100 family members and caveolin [71]. The oncogene c-Myc’s abnormal expression boosts the proliferative, invasive and migrative capabilities of HCC HepG2 cells, and therefore, a plasmid-based polymerase III promoter system was used to deliver and express siRNA to silence c-Myc in HepG2 cells. The results showed that the siRNA-based knockdown of c-Myc significantly decreased its expression in HepG2 cells by up to 85%. Consequently, a significant decrease in the migration, invasion and proliferation of the HepG2 cells was recorded [72]. Another study demonstrated that the overexpression of c-Jun, a proto-oncogene involved in mitogen-activated protein kinases (MAPK) signaling, was associated with Sorafenib HCC resistance; hence, when using a siRNA tool, c-Jun downregulation was correlated with significantly enhanced Sorafenib-induced tumor apoptosis [73]. Another oncogene in HCC is polycomb complex protein BMI-1 (Bmi-1), which facilitates DNA repair and promotes survival. A recent study correlated the resistance to Cisplatin treatment with elevated Bmi-1 expression levels. Cisplatin treatment alone or in combination with other drugs is used to combat HCC, especially in non-resectable or Sorafenib-refractory HCC [74,75,76]. On that basis, Li and colleagues developed, in 2019, a nanoparticle delivery system incorporating both nanoplatine cores and a calcium phosphate-coated siRNA targeting Bmi-1. The efficiency of the delivery and the silencing was tested first in vitro on HepG2 cells and then in vivo on a mice xenograft model. The results showed the highest tumor inhibition in the mice treated with nanoparticles loaded with calcium phosphate coated Bmi-1 siRNA and nanoplatine cores in comparison to that in the control groups [77].

Furthermore, a study showed that CD90^+^ Huh7 cells (HCC cell line) secreted exosomes harboring H19, a lncRNA, and once in the target cells, it upregulated the expression and release of VEGF, thus stimulating angiogenesis and the adherence of CD90^+^ Huh7 cells to endothelial cells [78], further stressing the importance of VEGF as a target for RNAi. A study was conducted in 2018 utilizing a galactose-derivative-modified nanoparticle harboring VEGF siRNA for an attempted knockdown. The results showed that VEGF siRNA loaded in asialoglycoprotein receptor-targeted nanoparticles silenced VEGF both in vitro and in vivo, demonstrated potent anti-angiogenic activity in HCA-1 tumors and suppressed primary HCC growth and distal metastasis [79]. A more recent study focused on attaining a synergistic effect by co-loading pH-sensitive liposomes loaded with a siRNA targeting VEGF, and Sorafenib. The system was tested in two-dimensional cultured HepG2 cells, three-dimensional HepG2 tumor spheroids and tumor regions of H22 tumor-bearing mice. The results showed a successful uptake of the system and decreased VEGF expression in all models along with an induction of apoptosis [80]. Moreover, and due to its role in drug resistance, a C-X-C chemokine receptor (CXCR) type-4-targeted lipid-based nanoparticle along with a modified antagonist of CXCR4, AMD3100, was designed to specifically deliver VEGF siRNA [81]. The results showed that the efficient downregulation of VEGF expression both in vitro and in vivo and together with Sorafenib led to synergistic tumor growth inhibition compared to Sorafenib only, suggesting that the use of siRNA in cancer therapy could increase drug efficacy [82].

### 4.2. Targeting Communication with Cellular Components

#### 4.2.1. Cancer-Associated Fibroblasts (CAFs)

CAFs are cells that trans-differentiate from different resident cells in the liver, mainly fibroblasts, but they can also derive from epithelial cells, endothelial cells, local mesenchymal cells, smooth muscle cells, pre-adipocytes and bone-marrow-derived progenitors [83,84,85,86].

Their trans-differentiation can be mediated by the tumor necrosis factor (TNF)-α and platelet-derived growth factor (PDGF) secreted by the macrophages [87]. HCC derives from a cirrhotic background, which involves a strong activation of fibroblasts, the main precursor of CAFs [88,89]. TNF-α was shown to upregulate the expression of the transcription factor apoptosis-antagonizing transcription factor (AATF) via its regulatory element site of sterol regulatory element-binding protein-1 in both HepG2 and Huh-7 cell lines via the siRNA targeting of the formerly mentioned regulatory element site. Furthermore, the knockdown of AATF in the HCC cell line QGY-7703 inhibited proliferation, migration, anchorage-independent growth, invasion and colony formation. A decrease in the tumorogenicity was also shown in the QGY-7703 xenograft model of NSG mice (non-obese diabetic severe combined immunodeficiency (SCID) gamma mice) harboring AATF knockdown. This study stresses the importance of TNF-α in HCC thriving [90].

On the level of tumor microenvironment priming, CAFs intercalate in the ECM and secrete ECM components. Among these, it is worth mentioning type 1 collagen fibers, fibronectin, tenascin and secreted protein acidic and rich in cysteine. Immune modulation-wise, CAFs secrete cytokines (interleukin(IL)-1, IL-6 etc.) and chemokines (monocyte chemoattractant protein-1 (MCP-1), C-X-C motif ligand (CXCL-)12 etc.). By the secretion of CXCL-12, CAFs also recruit endothelial progenitor cells to the tumor’s vicinity, thus supporting tumor vascularization [87]. To further support the tumor’s growth, CAFs produce and secrete growth factors (EGF, fibroblast growth factor (FGF), HGF and TGF-β) [91,92]. In addition, CAFs have been shown to secrete C-C motif ligand-(CCL)-2, -5 and -7 and CXCL-16, which in turn promote metastasis to bone, brain and lung in SCID mice via the activation of the TGF-β pathway [93], thus stressing the wide array of pathways activated by the crosstalk between CAFs and the tumor. These pathways are the focus of RNAi interventions.

#### 4.2.2. Endothelial Cells

Endothelial cells are important for the tumor as they are the nutrient and oxygen suppliers. In addition, they mediate crosstalk with the tumor via a change in the expression profile of receptors, rendering them responsive to signals derived from the tumor microenvironment and the tumor itself, while also secreting a variety of cytokines to communicate with the tumor [94]. The phenotype changes can be summarized by an upregulation of the expression of endoglin along with that of the various angiogenic receptors: VEGF receptor (VEGFR), EGFR, PDGF receptor (PDGFR) and CXCR [94]. RNAi-based gene silencing delivered by an optimized immunoliposome induced an effective EGFR gene knockdown in mice bearing orthotopic HCC, thereby showing the potential of this promising therapy [53]. The role of endoglin is prominent as it is activated by a variety of TGF-β superfamily members. It facilitates the extravasation of leukocytes into the site of inflammation and induces the “leaking” of chemotaxis factors into the bloodstream. Several studies have underlined the importance of IL-8-mediated inflammation in metastatic HCC through the activation of the transcription factor forkhead box C1 or via integrin αvβ3 [95]. The silencing of IL-8 with siRNA showed that it could be used to reduce the tumor metastasis mediated by IL-8 [96].

#### 4.2.3. Hepatic Stellate Cells (HSCs)

HSCs are normally non-proliferating cells residing in the liver, ready to be activated upon injury. These cells are primarily activated by signals from injured Kupffer cells, injured platelets or any other type of injured cell in the vicinity. In the context of HCC, HSCs are activated during the fibrogenesis process that precedes the tumorigenesis and continue to secrete ECM components. HSCs can be activated by HBV, HCV, cathepsins B and D, PDGF, TGF-β1, matrix metalloprotease (MMP)-9, JNK and insulin-like growth factor-binding protein 5 (IGFBP-5), and they can infiltrate into the HCC stroma. Once there, they stimulate tumor vascularization through the secretion of VEGF-A and MMP-2 [45]. They are known to secrete laminin-5, increase cytokine production, and exhibit liver-specific pericyte properties [97]. Various studies have been performed to stress the importance of the reciprocal crosstalk between HSCs and the tumor. A recent study showed that the crosstalk between HSCs and HCC via PDGF-β induced the increase in the expression of regenerating islet-derived protein 3 alpha (REG3A) in HCC cells. Furthermore, the silencing of REG3A via siRNA led to a decrease in the proliferation of LX-2 HCC cells when cocultured with the HSC cell line MH134. This is shown to be a result of the modulation of the p42/44 pathway [98].

A study showed an increase in the proliferation rate of rat HSCs following culturing with conditioned tumoral hepatocyte media, accompanied by an increase in α-smooth muscle actin expression and desmin, PDGFR and Gelatinase A secretion—markers of CAFs [99]. Another study showed the reciprocal priming of HSCs to the tumor. It showed that the conditioned media of HSCs increased the growth and invasiveness of HCC. Similar results were obtained upon the co-implantation of HSCs and HCC cells in nude mice, which appears to be as a result of activated NFκB and extracellular signal-regulated kinase (Erk) signaling pathways [100,101]. In that context, silencing Erk1 and Erk2 using siRNAs enhanced Fluorouracil sensitivity and increased Fluorouracil-induced apoptosis in the HCC HepG2 cell line, thus promoting chemosensitivity [102].

#### 4.2.4. Immune Cells

The landscape of the tumor microenvironment of HCC comprises a complex set of immune cells with their intricate cytokine and chemokine secretions. The immune responses sustained against HCC include the cytotoxic T-cells (CTLs), which produce perforin and granzymes to kill the cancer cells upon activation. The frequency of CTLs in the tumor’s vicinity is positvely correlated with survival. However, the crosstalk in the tumor’s vicinity between the tumor and these cells, along with effector molecules and immunosuppressive cells, namely T-regs, hinders the function of CTLs [43]. The tumor microenvironment plays a pivotal role in the immune evasion by establishing an immunosuppressive profile. The preferential activation of subsets of immune cells and their subsequent secreted chemokines and cytokines are the means for such a process. The crosstalk mediated between the immune cells and the tumor via the different secretory components plays a major role in the tumor‘s progression.

##### Macrophages

As HCC is a result of chronic inflammation, the persistent inflammatory signal drives the constant recruitment of monocytes into the inflammatory site along with an alteration in the bone marrow signal to favor the increased output of myeloid cells [103]. Thus, the pool of macrophages in the tumor site results from the invasion of circulating macrophages added to the pre-existing macrophages in the liver, termed Kupffer cells. The pool is heterogenous in terms of macrophage subtypes: M1, M2 or Tumor Associated Macrophages (TAMs) [104]. It has been shown that macrophages display a plasticity between the two major types of M1 and M2 macrophages. However, the line for discriminating between the two subtypes is not yet crystal clear. Principally, M1 macrophages (classically activated) are pro-inflammatory and are associated with anti-tumorigenic activity, whilst M2 macrophages (alternatively activated) are anti-inflammatory and favor tumorigenicity. The cell fate is decided by extrinsic factors ranging from growth factors, cytokines and chemokines to microenvironment stress. M1 macrophages are stimulated by interferon (IFN)-γ along with a TLR agonist such as lipopolysaccharide. M2 macrophages, on the other hand, are stimulated by IL-4, IL-10 and IL-13. The differentiation implicates phenotypical, genetic, epigenetic, metabolic and secretome changes. Of major importance in the context of cancer is the profile of the secretion of each macrophage subtype. On the level of M1 macrophages, they exhibit a typical pro-inflammatory cytokine and chemokine profile: TNF- α, IL-1, IL-6, IL-12, IL-18, IL-23, MCP-1, CXCL9 and CXCL10. As for M2 macrophages, they are known for the secretion of IL-10, IL-12^low^, CCL17, CCL22 and CCL24 [104,105]. M2 macrophages are active effectors in the context of HCC. A clinical study in 2014 showed a poor prognosis for patients exhibiting an increased expression of CD-163 and Scavenger A macrophages (markers for M2 macrophages). This was accompanied by increased tumor nodules and venous infiltration in HCC, along with an increased epithelial–mesenchymal transition potential via M2 macrophage CCL22 secretion [106]. Another study showed that M2 macrophages accumulated more in Sorafenib-resistant HCC tumors than in Sorafenib-sensitive ones, and confer Sorafenib resistance by secreting HGF, which sustains tumor growth and metastasis by the activation of the HGF/c-Met, ERK1/2/MAPK and PI3K/AKT pathways in tumor cells [107].

Several targeting mediators of tumor cells are secreted by macrophages and have been studied in the development and progression of HCC. In the context of targeting MAPK signaling, which is overexpressed in HCC and associated with tumor growth, the downregulation of mitogen-activated *protein* kinase kinase kinase kinase 4 (MAP4K4) using shRNA led to reduced cell proliferation, S-phase cell cycle blockade and increased tumor apoptosis [108]. Another signaling pathway targeted by RNAi is HGF/c-Met. A study showed that small ubiquitin-like modifier (SUMO) specific protease 1 silencing resulted in a downregulation of HGF-induced proliferation and migration of HCC cells through effects on the HGF/c-Met pathway [109]. Additionally, in order to inhibit the proliferation of HCC cell lines (HepG2 and Huh7), siRNA targeting a tyrosine kinase receptor known as macrophage-stimulating protein receptor was shown to efficiently suppress tumor cell migration and invasion and enhance apoptosis by activating cleaved caspase-3 and poly (ADP-ribose) polymerase through the modulation of the Akt, c-Raf and ERK signaling pathway [110]. A study in 2019 by Zhang and colleagues evaluated the expression of sirtuin 6 (SIRT6) in a variety of HCC cell lines and its effect on the Erk1/2 signaling pathway favoring proliferation and inhibiting apoptosis. The results showed an elevated expression of SIRT6 in nine HCC cell lines in comparison to in a normal liver cell line. Moreover, the knockdown of SIRT6 via a siRNA approach in the Huh-7 cell line resulted in a decrease in the proliferation rate along with an increase in the apoptosis rate, with a downregulation of B-cell lymphoma 2 (Bcl-2) and an upregulation of Bcl-2-associated X protein (Bax) and cleaved-caspase 3. This was accompanied by a decrease in the phosphorylation of Erk1/2 [111]. Epithelial cell transforming sequence 2 (ECT2) has been shown to be implicated in early HCC recurrence via the activation of Rho/Erk signaling. The downregulation of ECT2 by siRNA entailed the suppression of Erk, thereby enhancing apoptosis and reducing the metastatic ability of HCC cells [112]. Further focusing on Rho, Ras homolog family member C (RhoC) overexpression and the metastatic potential of HCC have been correlated with the enhanced invasion and migration of HCC cells. The inhibition of RhoC resulted in the inhibition of invasion and migration without reducing cell viability in HCCLM3 cells. In addition, the silencing of RhoC expression in an HCC metastatic mouse model significantly inhibited tumor metastasis [113].

Another subset of macrophages termed “TAMs” was found in the vicinity of HCC tumors. Studies have shown that the presence of TAMs in the tumor’s vicinity correlates with poor prognosis in HCC. These cells exhibit an M2-like phenotype and express both M1 and M2 macrophage hallmarks. They secrete pro-inflammatory cytokines, e.g., IL-1β, IL-6, IL-23 and TNF-α. A recent study demonstrated that the interaction of TNF-α and angiotensin II in HCC cells could enhance tumor proliferation, migration and invasion via the regulation of G protein-coupled receptor kinase 2 (GRK2). Therefore, GRK2 siRNA was used to examine the molecular interactions of TNF-α in tumor growth, and the obtained results suggested that TNF-α could serve as a new potential therapeutic target in HCC [114]. TAMs also secrete a variety of growth factors such as TGF-β, VEGF, FGF, PDGF, angiogenic factor thymidine phosphorylase, angiogenesis-modulating enzyme cyclooxygenase-2, MMP-9 and MMP-12 [105,115]. On the level of immune response modulation, TAMs have been shown to suppress CD4^+^CD25^−^ T cells, activate T-regs and contribute to the expansion of Th-17 cells, which, in turn, favor the expression of the immune-suppressing antigens PD-1, CTLA-4 and glucocorticoid-induced TNF receptor family-related (GTIR) [116,117]. Moreover, TGF-β in the tumor microenvironment triggers the expression of TIM-3 on the surface of TAMs and subsequent IL-6 secretion; the TAM-derived IL-6 further activates the IL-6/signal transducer and activators of transcription (STAT3) pathway in the tumor, sustaining survival and proliferation [43,118]. When HCC cell lines (SMMC7721 and QGY-7703) were transfected with siRNA targeting STAT3 and AKT2, a significant decrease in the mRNA level of AKT2 and STAT3 was observed. Furthermore, nude mice were used to verify the correlation, and a decreased ability for HCC cell proliferation, migration and invasion has been since concluded [119].

##### Neutrophils

Neutrophils in the vicinity of a tumor are termed tumor-asociated neutrophils (TANs). As is the case with macrophages, TANs in the tumor microenvironment exhibit two polarizations: N1 neutrophils that are said to be anti-tumorigenic and N2 neutrophils that are said to be pro-tumorigenic [120]. Their activation is governed by type 1 IFNs and TGF-β. Their main role in tumors is to suppress CD8^+^ T-cells, thus helping the tumor evade the immune response. Nitric oxide production by TANs, induced by TNF-α in the tumor microenvironment, promotes CD8^+^ T-cell appoptosis [121]. In the context of HCC, TANs exhibit the same role as in different cancers, associating with poor prognosis and driving tumor progression. The neutrophil–lymphocyte ratio (NLR) is of great significance in patients subject to immunotherapy (Programmed cell death 1 (PD-1)/PDL-1) and correlates with tumor progression [122,123]. On the clinical level, NLR has been shown to be an indicator of survival after hepatectomy [124,125]. Recent studies have shown that the accumulation of TANs via hypoxia-inducible factor (HIF)-1/CCL5 upregulation in NASH drives the initiation and progression into HCC [126]. While in the tumor’s vicinity, TANs secrete chemokines such as CCL2 and CCL17, which, in turn, recruit TAMs and T-regs, thus contributing to HCC progression, metastasis and resistance to Sorafenib treatment [127]. Interestingly, the downregulation of CXCL5—a direct chemoattractant for neutrophils—by shRNA in HCC cells reduced tumor growth, metastasis and intratumoral neutrophil infiltration [128].

##### Dendritic Cells

Dendritic cells (DCs) in a healthy liver play the role of bridging the innate and adaptive immunity as antigen-presenting cells (APCs) as well as of instructing lymphocytes. The most prominent role of DCs in HCC is mediating immune tolerance. Thus, the crosstalk between DCs, the tumor and its tumor environment is mediated by the secretion of various cytokines, leading to immune tolerance and tumor progression. At first hand, the activation of DCs is mediated by IFN-γ secretion by the tumor or effector cells in the vicinity. Upon activation, DCs tend to secrete IL-10 and IL-12. IL-12 leads to the impaired activation of allogenic T-cells. IL-10 depotentiates the immune response against the tumor and excludes APCs from the tumor mass [129]. It is important to mention that the DCs are emerging targets for immune therapy in the context of HCC, thus further stressing the fact that the crosstalk between DCs, immune cells and the tumor is crucial for tumor progression [130]. A study demonstrated that the anti-tumor effect of IFN-γ, which mediates cell death by autophagy, can be regulated by the modulation of interferon regulatory factor (IRF)-1 by shRNA [131]. On another note, IFN-stimulated gene 15 (ISG15) is a ubiquitin-like molecule that has been identified as an intrinsic actor that elicits HCC tumorigenesis and metastasis; the former is overexpressed in HCC patients. Using cell lines and a xenograft model, the siRNA-mediated knockdown of ISG15 was shown to significantly inhibit tumor growth [132].

##### Natural Killer Cells

Natural killer (NK) cells are prominent in the liver and are the first responders against viral infections (HBV and HCV). NK cells are also in charge of maintaining proper immune function as they regulate the tuning of the immune response between the defensive and tolerance modes [105,133]. The inhibitory function of NK cells in HCC supports the tumor thriving. Growing evidence suggests that the hypoxic conditions inducing the activation of HIF-1α along with immune modulators in the tumor microenviroment disrupt the regulating ability of NK cells, resulting in the exhaustion of the anti-tumor response and poor prognosis [134]. Interestingly, the RNAi-mediated suppression of HIF-1α expression in the liver inhibited metastatic tumor growth in the hepatoma cell line (SMMC-7721) and tumor-bearing mouse liver [135,136]. NK cells can be inhibited by HCC cells as HCC cells express major histocompatibility complex (MHC) class I polypeptide–related sequence A, a specific NK cell ligand that inhibits NK cell interaction [91,105]. Another mechanism of the depotentiation of NK cells by HCC cells is the impairment of IL-12 secretion by DCs by HCC cell-derived α-fetoprotein (AFP). A more direct effect of AFP has been shown on NK cells and is dictated by the time frame [137,138]. T-regs play a role in the attenuation of the NK cell’s function, either by the release of cytokines such as IL-8, TGF-ß1 and IL-10—which then decrease the expression of NK receptors’ ligands on HSCs, preventing them from binding to the NK group 2D receptor on NK cells—or by their competition with NK cells for the available IL-2 in the tumor microenvironment [139]. Moreover, the attenuation of the production of TNF-α and IFN-γ by NK cells mediated by CAF-derived indoleamine-pyrrole 2,3-dioxygenase and prostaglandin E2 has been shown to be among the reasons for sustained fibrosis in HCC and immune cell evasion [140].

## 5. Challenges and Future Perspectives

HCC is a primary type of liver cancer with a high mortality rate and a poor prognosis. It has varying advancement and occurrence rates epidemiologically according to the environmental factors in each region of the world, stressing a vast group of risk factors [141]. To date, the treatment options for this type of cancer are Sorafenib, Lenvatinib, Regorafenib and Cabozantinib, which improve patient prognosis but with the cost of side effects and deterioration in the quality of life. Hence emerges the need for alternative treatment options. Research in this field has increased dramatically, with the search for other first-line and second-line treatment options exploiting various angles: immunotherapy, selective tyrosine kinase inhibitors, anti-angiogenic agents or combination therapy, among others. Aligned with these current developments, a first-in-human study of the small double-stranded activating RNA oligonucleotide MTL-CEBPA has shown that the pre-treatment of the HCC tumor microenvironment with MTL-CEBPA renders it more susceptible to the effects of established anti-HCC therapies, which shows the great potential of innovation in HCC treatment [142]. As extensive as the search for alternative treatments is, another approach is exploiting RNAi for a highly targeted therapy, targeting the impairments of the different molecular pathways in HCC exacerbated by the extensive crosstalk with the tumor microenvironment actors, as described in this review. However, such an approach faces many difficulties that need to be addressed. As previously explained, the definitive actor and director of such an approach is the nucleic acid composed of either an siRNA, shRNA or miRNA.

This tool confers the advantage of designing an intervention down to the gene level. si/sh/miRNA sequences can be customized to target a specific gene or even a gene corresponding to a specific isotype of the protein. Part of the equation in RNAi is the specificity of the effector molecule and its “exclusive” effect on the set target. An extended analysis should be done on the off-target effects of a given RNAi tool prior its use to provide a better picture of the biosafety. The off-targets of such an approach can be predicted by in silico methods, unlike those of small-molecule drugs, where the undesired effects often remain unraveled until further in vitro and in vivo testing. Moreover, a study published in 2019 showed that multiple cancer drug candidates kill tumor cells through off-target effects instead of by interacting with their intended molecular targets. They showed by the clustered regularly interspaced short palindromic repeats (CRISPR)-Cas9 deletion of a target that small-molecule drugs kill the target-KO cells as efficiently as the wild-type cells, suggesting that not on-target but rather off-target interactions are frequently the real mechanism by which small-molecule drugs block cancer growth [143]. On the other hand, the in silico study of the sequences of RNAi, via “NCBI blasting” for instance, gives an overview of the possible off-target effects via a simple blasting of the sequence of interest against the whole genome of humans—or any other organism. The results show a list of the various annealing possibilities, which could be tested and controlled prior to functional assessment. One additional advantage is the possibility of designing sequences that cross-react among species. Exploiting the “NCBI blasting” in silico system and blasting the sequence of interest against rodent and human genomes could result in a candidate sequence that can be used in both. This is one of the great advantages of utilizing RNAi as a treatment option, as the molecule tested preclinically in an in vivo rodent model is exactly the same as the one that will be used in humans, which could minimize the variables to be controlled in clinical trials.

The effectiveness of RNAi is dictated by the ability of the si/sh/miRNAs to reach their targets. The obstacles faced in an in vitro system are mainly the cell membrane and the endosomal escape. The selectivity of the cell membrane hinders the internalization of the sequences introduced; thereafter, the endosome poses a great threat to the integrity of the sequences, as they could be degraded by endosomal enzymes. Moreover, in a complex in vivo system, the obstacles are exacerbated due to the complexity of the human body [144]. Great progress has been made during the past decade regarding the delivery of RNAi. There are still many challenges for extrahepatic organs, where RNAi delivery by oral or intravenous administration is still limited due to vascular barriers. However, the liver is the prime organ target for systemically delivered RNAi due to its relatively open vasculature. Furthermore, researchers are taking advantage of the expression of the asiaglycoprotein receptor on the surface of hepatocytes to ameliorate the delivery to the liver [145]. Recently, RNAi administration during liver machine preservation was proposed. This technique harbors many advantages as it can further increase the targeted delivery to the liver with lower RNAi doses and, subsequently, at a lower cost while avoiding side effects on other organs [146]. These approaches may open up novel possibilities for RNAi therapeutics in the HCC field.

Various studies in the RNAi field have focused on modulating the carrier to increase the efficiency of the delivery by regulating the variables of size, charge, pH, composition, etc. Additionally, the development of transport carriers capable of selective siRNA delivery is particularly important when targeting the tumor microenvironment. A liver with HCC tumors is known to contain an abundant population of immune cells with immunosuppressive functions in the tumor microenvironment. RNAi therapeutics that could restore the anti-tumor immune response in HCC are needed, but the development of transport carriers capable of the selective delivery of siRNA to specific immune cells remains challenging. The effect of a siRNA could be cell-dependent, as different cells would express different isoforms and/or amounts of the mRNA of a specific target. As reviewed in this article, the expression level of a given protein may have a drastic effect on the secretory profile of the cell—taking into account the different subpopulations of T-cells, macrophages and neutrophils, each with their own transcriptomic profile. Thus, the effect of an siRNA targeting a specific protein could be contradictory if it not delivered to the right cell population and/or subpopulation. Moreover, it is important to keep in mind that some carriers, without taking into account the siRNA carried, can modulate the immune system, especially the functions of APCs [147].

One carrier of natural origin, which could avoid the hindrances of synthetic nanoparticle carriers, is exosomes. As described in the article, these extracellular vesicles serve as shuttles of nucleic acids and proteins that are the means of intercellular communication between the various cell types. The notion of exosomes became clearer over time, as they were once said to be “waste disposal vehicles” before their crucial role in cell communication was unraveled. Exosomes are of particular interest in immune cells’ communication as they are naturally excreted from several immune cells including dendritic cells, T-cells, mastocytes and B-cells [148], and have been reported to be a key effector of inflammation and tumor microenvironment communication [149]. Exosomes have been shown to activate or suppress innate immunity and regulate the TLR/NFkB signaling pathway. Interestingly, exosomes have been exploited as ways of administering epitopes for anticancer vaccination. Moreover, exosomes have become incorporated in theragnostic approaches and facilitated the access to biomarkers to follow the evolution of the disease through miRNAs carried in exosomes, especially in lung cancer, liver cancer, gastrointestinal cancer, pancreatic cancer, melanoma, breast cancer, ovarian cancer and prostate cancer [63]. During the past years, exosomes have been studied as a new natural vector for shuttling RNAi molecules due to their high biocompatibility for various reasons: (i) exosomes are delimited by a lipid bilayer, which confers a great advantage when fusing with other cell types; (ii) exosomes can utilize a receptor-mediated endocytosis mechanism for cell entry, thus conferring a specific cell targeting advantage; and (iii) exosomes have been shown to traverse the bloodstream easily, establishing metastatic niches for cancer, thus avoiding the hindrances of the vascular barriers facing other RNAi carrier particles [150,151]. In that context, Morishita and colleagues performed an in vivo study on the bioavailability of exosomes. The results showed that exosomes were mostly concentrated in the liver, spleen and pancreas in addition to other abdominal organs and the lungs [152], shedding light on a possible enhancement of liver targeting. However, loading cargo into exosomes remains challenging [149], and developing new strategies to vectorize RNAi molecules through exosomes could be rendered beneficial in exosome-RNAi therapy. In the meantime, new RNAi vectors developed based on natural exosomes are under investigation and have shown promising results for efficiently delivering RNAi molecules. This is highlighted by the study of Lunavat and colleagues where they delivered c-Myc specific siRNA into cancer cells through exosome-mimetic nanovesicles [153]. More recently, the study of Zhou and colleagues has shown that the delivery of Kirsten Rat Sarcoma viral oncogene homolog siRNA with internalizing arginylglycylaspartic acid (RGD) exosomes efficiently inhibits tumor growth in a mouse model of lung cancer [154].

As complex as the optimization process is, RNAi still serves as a source of hope and a valid candidate for a treatment option for HCC. We are optimistic that with the advancements in the scientific approaches and the continuous unraveling of the molecular impairments that drive HCC, an RNAi approach with the correct combination of a carrier and a nucleic acid component will one day significantly improve the outcomes of HCC patients.

## Figures and Tables

**Table 1:** Clinical trials of RNAi-based therapeutics for solid tumors including hepatocellular carcinoma (HCC). EPHA2: ephrin type-A receptor 2, PLK1: polo-like kinase 1, STMN: stathmin, EGF: epidermal growth factor, KSP: kinesin spindle protein, GMSF: granulocyte-macrophage colony-stimulating factor.

Drug Name	Target	Phase	Company	NCT Reference	Status
siRNA-EphA2-DOP C	EPHA2	Phase I	MD Anderson	NCT01591356	ongoing
TKM-PLK1/TKM-080301	PLK1	Phase I/II	Arbutus	NCT02191878	completed
pbi-shRNA STMN	STMN	Phase Ib/2	Gradalis	NCT01505153	completed
ALN-VSP02	VEGF and KSP	Phase I	Alnylam	NCT01158079	completed
Fang	Furin and GMSF	Phase I	Gradalis	NCT01061840	completed
DCR-MYC	MYC	Phase I	Dicema	NCT02314052	terminated
MRX34	miR-32 mimic	Phase I	Mirna Therapeutics	NCT01829971	terminated

## Abbreviations

HCC	hepatocellular carcinoma
RNAi	RNA interference
HCV	viral hepatitis C
HBV	viral hepatitis B
NASH	non-alcoholic steatohepatitis
PDL-1	anti-programmed death-ligand1
VEGF	anti-vascular endothelial growth factor
mRNA	messenger RNA
ncRNA	non coding RNA
dsRNA	double-stranded RNA
TLR	Toll-like receptor
miRNA	microRNA
shRNA	short hairpin RNA
siRNA	small interfering RNA
nt	nucleotide
pri-miRNA/shRNA/siRNA	primary–miRNA/shRNA/siRNA
pre-miRNA/shRNA/siRNA	precursor-miRNA/shRNA/siRNA
RISC	RNA induced silencing complex
PEI	polyethylenimine
EPHA2	ephrin type-A receptor 2
PLK1	polo-like kinase 1
STMN	stathmin
KSP	kinesin spindle protein
GMSF	granulocyte-macrophage colony-stimulating factor
TGF	transforming growth factor
ECM	extracellular matrix
CAFs	cancer associated fibroblasts
HSCs	hepatic stellate cells
TAMs	tumor associated macrophages
TANs	tumor-associated neutrophils
DCs	dendritic cells
T-regs	T-regulatory cells
HS	heparin Sulfate
GPC3	glypican 3
EGF	epidermal growth factor
HGF	hepatocyte growth factor
EGFR	epidermal growth factor receptor
NET-1	neuroepithelial cell transforming-1
EMS1	*gene encoding protein: leucine-rich repeat receptor protein kinase EMS1*
MIF	migration inhibition factor
CCN	cellular communication network factor
CYR61	cysteine-rich angiogenic protein 61
CTGF	connective tissue growth factor
NOV	nephroblastoma overexpressed
WISP-1	Wnt1-Inducible Signaling pathway proteins
NFκB	nuclear factor kappa B
MSCs	mesenchymal stem cells
TIM-3	T-cell immunoglobulin and mucin domain-containing molecule-3
LAG3	lymphocyte-activation gene 3
CTLA-4	cytotoxic T-lymphocyte-associated protein-4
MAPK	microtubule associated protein kinase
Bmi-1	polycomb complex protein
CXCR	C-X-C chemokine receptor type
TNF	tumor necrosis factor
PDGF	platelet-derived growth factor
AATF	apoptosis-antagonizing transcription factor
NSG	non-obese diabetic severe combined immunodeficiency gamma mice
SCID	severe combined immunodeficiency
IL	interleukin
MCP-1	monocyte chemoattractant protein-1
CXCL	C-X-C motif ligand
FGF	fibroblast growth factor
CCL	chemokine C-C motif ligand
VEGFR	vascular endothelial growth factor receptor
PDGFR	platelet-derived growth factor receptor
MMP	matrix metalloprotease
GTIR	glucocorticoid-induced TNF receptor family-related
IGFBP-5	insulin-like growth factor-binding protein 5
REG3A	regenerating islet-derived protein 3 alpha
Erk	extracellular signal-regulated kinases
CTLs	cytotoxic T-cells
IFN	interferon
MAP4K4	mitogen-activated protein kinase kinase kinase kinase 4
SUMO	small ubiquitin-like modifier
SIRT6	sirtuin 6
Bcl-2	B-cell lymphoma 2
Bax	Bcl-2-associated X protein
ECT2	epithelial cell transforming sequence 2
RhoC	Ras homolog family member C
GRK2	G protein-coupled receptor kinase 2
GTIR	glucocorticoid-induced tumor necrosis factor receptor
STAT3	Signal Transducer and Activators of Transcription
NLR	neutrophil–lymphocyte ratio
PD-1	programmed cell death protein-1
HIF-1	hypoxia-inducible factor
APCs	antigen-presenting cells
IRF	interferon regulatory factor
ISG15	IFN-stimulated gene 15
NK	natural killer
AFP	α-fetoprotein
CRISPR	clustered regularly interspaced short palindromic repeats
RGD	arginylglycylaspartic acid
